# Machine learning driven design and optimization of a compact dual Port CPW fed UWB MIMO antenna for wireless communication

**DOI:** 10.1038/s41598-025-98933-w

**Published:** 2025-04-22

**Authors:** Jayant Kumar Rai, Swati Yadav, Ajay Kumar Dwivedi, Vivek Singh, Pinku Ranjan, Anand Sharma, Somesh Kumar, Stuti Pandey

**Affiliations:** 1 Department of Electronics and Telecommunication, RKR Government Polytechnic, Janjgir Champa, Chhattisgarh, India; 2 Department of Electrical and Electronics, College of Engineering, COER University, Uttarakhand, India; 3 Department of Electronics and Communication Engineering, Nagarjuna College of Engineering and Technology, Bengaluru, Karnataka India; 4 Department of Electrical and Electronics Engineering, ABV Indian Institute of Information Technology and Management, Gwalior, Madhya Pradesh India; 5 Department of Electronics and Communication Engineering, Motilal Nehru National Institute of Technology Allahabad, Prayagraj, Uttar Pradesh India; 6 Department of Artificial Intelligence & Machine Learning, School of Computer Science & Engineering, Manipal University Jaipur, Jaipur, India

**Keywords:** Coplanar waveguide (CPW), MIMO, Machine learning, Ultra-Wide band (UWB), Electrical and electronic engineering, Electronics, photonics and device physics

## Abstract

In this article, a compact dual port Multiple Input Multiple Output (MIMO) Coplanar Waveguide (CPW) fed Ultra-Wideband (UWB) antenna for the next generation wireless communication using Machine Learning (ML) optimization is presented. It is designed on an FR4 epoxy substrate of 16 × 30 mm^2^ with a thickness of 1.6 mm. A bandwidth of 8.7 GHz (2.78–11.48 GHz) is achieved. It is used for 5G New Radio Bands (n78/n46/n47/n77/n48/ n79/n96), Wi-Fi 5, DSRC, Wi-Fi 6, and Vehicle to Infrastructure (V2I), Vehicle to Vehicle (V2V), and Vehicle to Network (V2N) in the entire operating band. The proposed antenna is optimized through the different ML algorithms Artificial Neural Network (ANN), Extreme Gradient Boosting (XGBoost), Random Forest (RF), K-Nearest Neighbor (KNN), and Decision Tree (DT). The DT ML algorithms provide a higher accuracy of 99.92% compared to the remaining ML algorithms. A test and fabrication of the suggested antenna is also done. The findings showed that there was a good correlation between measurement and simulation data for several parameters, including S-parameters, radiation patterns, and MIMO parameters like diversity gain (DG), channel capacity loss (CCL), mean effective gain (MEG), envelope correlation coefficients (ECC), and total active reflection coefficients (TARC). Hence, it is suitable for next-generation wireless communication.

## Introduction

Nowadays, Multiple Input Multiple Output (MIMO) wireless communication is the fastest-growing technology that fulfills all the requirements of communication systems such as enhancing the channel’s capacity. To design a MIMO antenna, the strong isolation between the two antenna is essential and the spacing between the two antenna components isthe key parameter that decides the isolation between the components. Therefore, the main challenge of antenna designers is to design small MIMO antennas with high isolation. In MIMO antennas Coplanar Waveguide (CPW) fed provides several advantages over the microstrip line such as its fabrication is easy and allows it easier to combine with monolithic microwave integrated circuit (MMIC) equipment. CPW-Fed also achieves lower dispersion and low radiation loss. Some of the previously presented CPW-fed MIMO antenna are shown in^1,2^. UWB antenna use has increased significantly because it receives low power consumption. In addition, the UWB system specifies other benefits associated with higher data bandwidth that supports various applications like medical imaging systems, mobile systems, and vehicle radar systems^3,4^. A CPW Fed has several advantages over microstrip Feds, including the simplicity of attaching electronic components due to its coplanar design, no need to drill through the substrate, and a smooth transition to the slot line. As a result, these Feds are chosen for applications when space is limited. Several CPW-fed antennas for UWB applications have been presented by earlier investigations^5–14^.

When designing an antenna, machine learning (ML) is an accurate and highly suggested optimization approach. ML finds applications across various domains, including automated translation, image processing, collaborative filtering, time series forecasting, and categorization. In antenna design, the configuration is inherently dependent on geometric factors. Optimization, in this context, involves the manipulation of antenna parameters to achieve a desired and refined design outcome. To get the required parameters, optimization will be carried out by altering the dimensions of certain antenna characteristics. Trial-and-error has always been used to carry out the optimization process. This process takes a lot of time. ML techniques may be used to estimate the characteristics of several antenna modules quickly and reliably. ML will analyze data to detect undiscovered mathematical relationships, link input, and output behaviors, and make predictions. Therefore, using previously adopted methodsis not optimal in the antenna design^15–22^. To solve this issue in this paper, we have investigated the various ML algorithms for designing the antenna to improve performance.

A few of the CPW-Fed MIMO antennas designed in the past few years are compared in Table 1. In^23^an inverted ‘A’ and ‘Y’ shaped structure with small extended stubs is used to achieve good isolation in the designed MIMO antenna. In^24^, A flexible CPW-fed MIMO antenna with fence-shaped decoupling branches to attain high isolation is presented. In^25^to get good isolation between the two antennas a well-designed meta-material structure is reported. In^26^, Two monopole antenna components placed perpendicularly to each other with a stub placed in the middle of the radiating element to increase the bandwidth and isolation is designed. In^27^ an antenna with two radiating components placed edge to edge with a strip in the center to provide high isolation is presented. The proposed CPW-fed MIMO antenna has the following attractive characteristics:

1. It provides an Ultra-Wide Band (UWB) between 2.78 GHz and 11.48 GHz. The bandwidth is 8.7 GHz and the percentage of impedance bandwidth is 122.02%.
2. It is very compact in size. The size of the antenna is 16 × 30 mm^2^.
3. It is optimized through Artificial Neural Network (ANN), Extreme Gradient Boosting (XGBoost), Random Forest (RF), Decision Tree (DT) and K-Nearest Neighbor (KNN) ML algorithms. The DT ML algorithms provide a higher accuracy of 99.92% compared to the remaining ML algorithms.
4. It is used for 5G New Radio Bands (n77/n46/n78/n47/n48/n79/n96), DSRC, Wi-Fi 5, Wi-Fi 6, and V2 V, Vehicle to Network V2 N, and V2I in the entire frequency range.
5. Throughout the whole working band, there is more than 12 dB of isolation between the ports.

**Table 1:** Comparison of CPW fed MIMO antennas.

Ref.	Size (in mm^3^)	Frequency range (GHz)	Isolation (-dB)	ECC	DG	Machine Learning
^23^	47 × 32 × 0.8	3–7.70	< 20	< 0.04	9.98	No
^24^	56 × 30 × 0.1	3–15.7	< 14.5	< 0.005	9.998	No
^25^	48 × 35 × 1.6	2–18	< 10	< 0.1	9–10	No
^26^	27 × 27 × 0.8	2–11	< 15	< 0.02	NA	No
^27^	36 × 36 × 1.6	3.1–14.9	< 13.7	< 0.01	9.9	No
**Proposed Antenna**	**16 × 30 × 1.6**	**2.78–11.48**	**< 12**	**< 0.25**	**9.998**	**Yes**

## Antenna configuration and analysis

### Antenna configuration

**Fig. 1:**
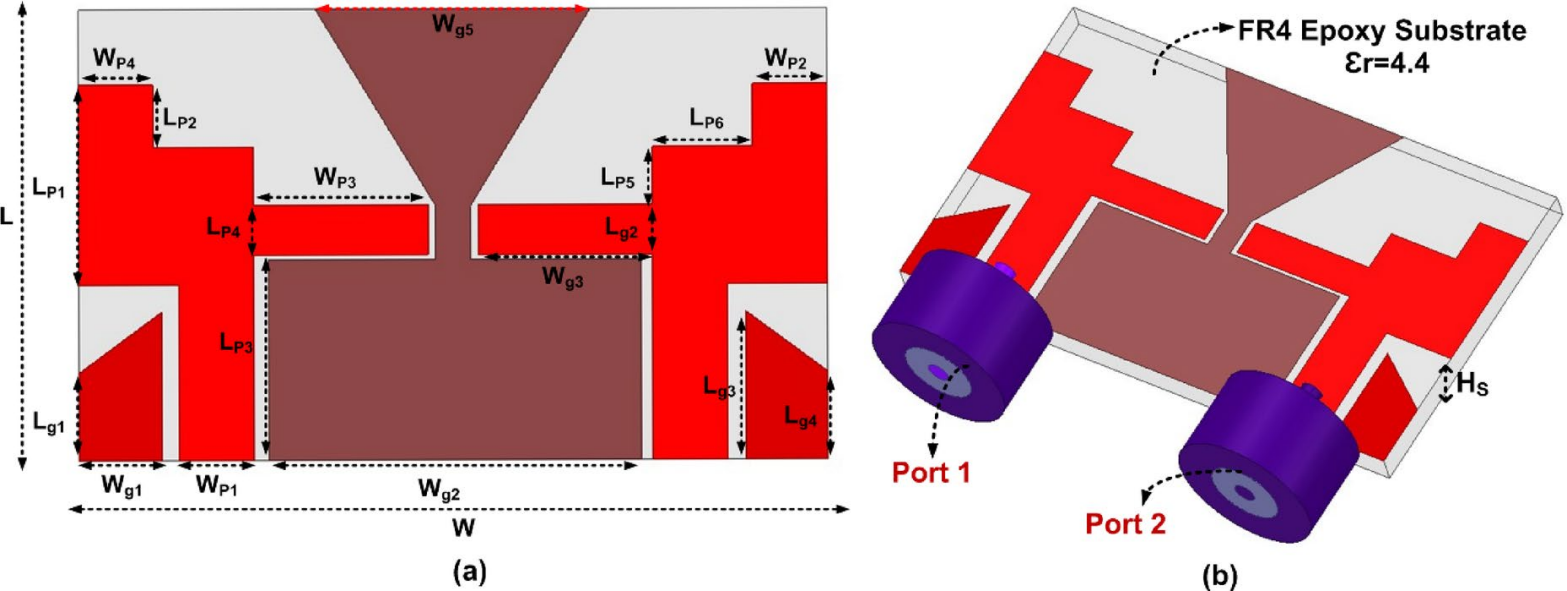
Layout of the proposed MIMO antenna (L = 16, L_P1_ = 8, L_P2_ = 2.5, L_P3_ = 7, L_P4_ = 2, W_P1_ = 3,W_P2_ = 4, W_P3_ = 7, W_P4_ = 3, W_P5_ = 4, W = 30, L_g1_ = 2.3, L_g2_ = 8, L_P11_ = 14.9, L_g3_ = 2, L_g4_ = 5.926, W_g1_ = 3.3, W_g2_ = 14.8, W_g3_ = 6.7, W_g4_ = 1.4, W_g5_ = 11).

**Fig. 2:**
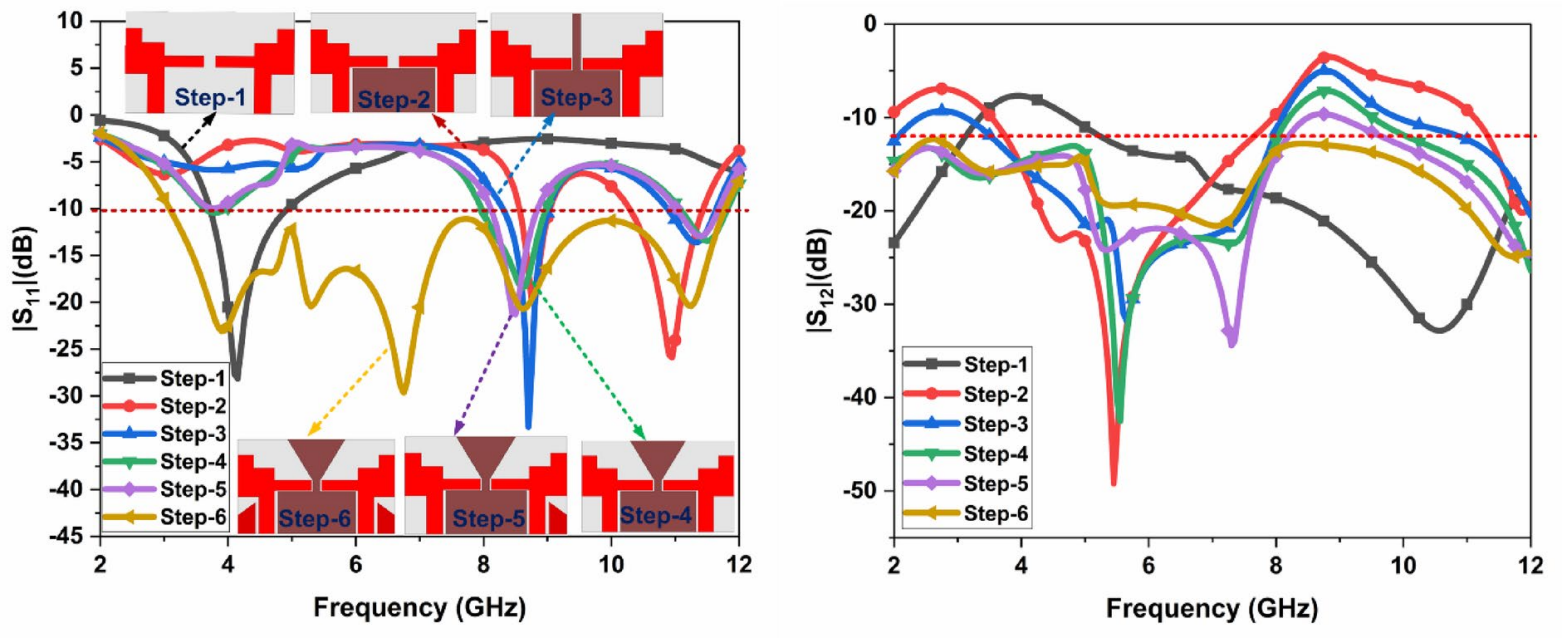
Various design steps of the proposed antenna.

**Fig. 3:**
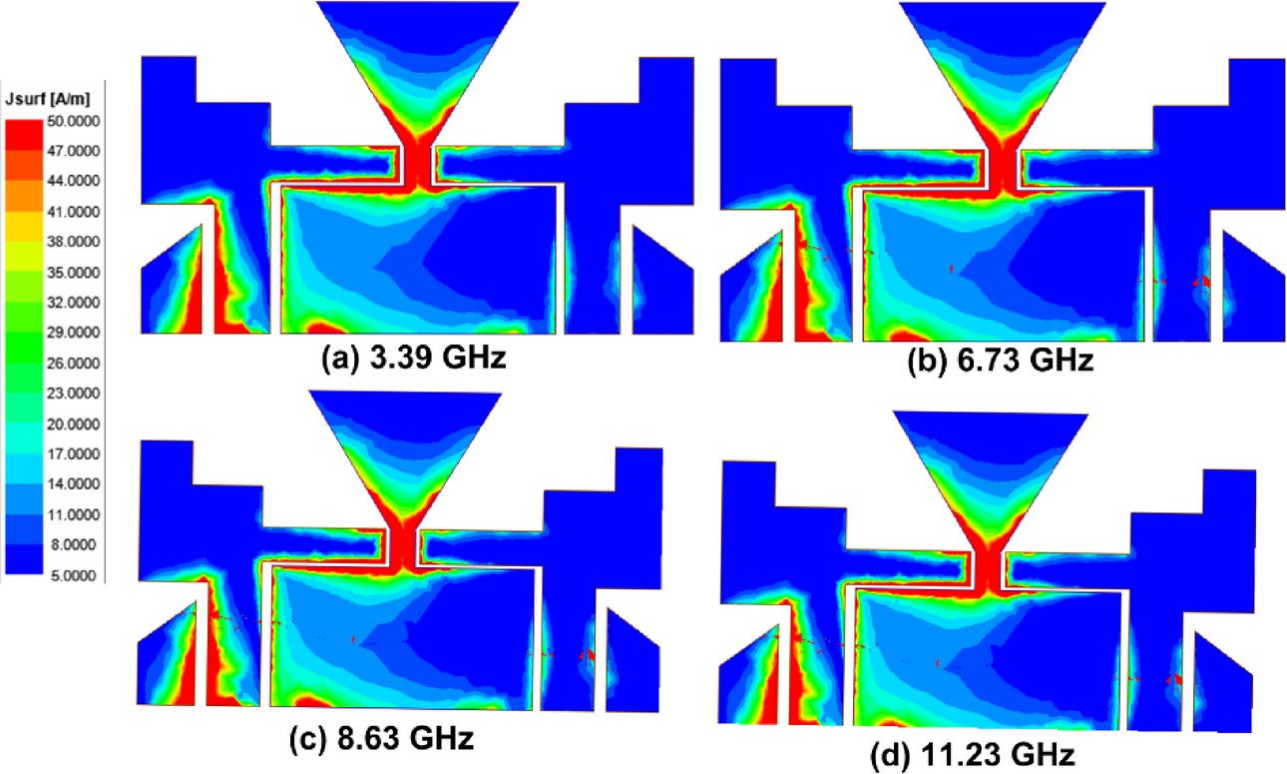
Surface current distribution at various frequencies.

The arrangement of the suggested CPW Fed MIMO antenna is illustrated in Fig. 1. The proposed antenna is designed on an FR4 epoxy substrate ($\:{\in\:}_{r}$ = 4.4 and loss tangent tanδ = 0.02) with a thickness of 1.6 mm. The overall size of the antenna is 16 × 30 mm^2^. Two ladder-shaped structures extended towards the outward side are designed as the radiating patch on the substrate and to provide the higher isolation among the two antennas a triangular ground plane is created between the radiators and two different ground planes are created on the opposite side of the radiator. CPW Fed is used to feed the two radiators. The gap between the Feedline and the ground outwards is kept at 0.6 mm and 0.7 mm from the ground towards the center. Figure 2 depicts the various phases of the design of the suggested antenna. and the FR, BW, and % impedance BW improvement are given in Table 2. In Step 1, the MIMO antenna provides a single band from 3.73 GHz to 4.93 GHz has a maximum $\:{S}_{11}$of −28.61. In Step 2, the MIMO antenna provides a dual-band from 8.56 GHz to 8.96 GHz and 10.3 GHz to 11.43 GHz has a maximum $\:{S}_{11}\:$of −20.34 and − 26.05. In Step 3, the MIMO antenna provides a single band from 8.33 GHz to 8.96 GHz has a maximum $\:{S}_{11}$of −33.3. In Step 4, the MIMO antenna provides a dual-band from 8 GHz to 8.96 GHz and 11.03 GHz to 11.76 GHz has a maximum $\:{S}_{11}\:$of −18.57 and − 13.45. In Step 5, the MIMO antenna provides a dual-band from 8.13 GHz to 8.83 GHz and 11.03 GHz to 11.66 GHz has a maximum $\:{S}_{11}\:$of −21.50 and − 12.96. In Step 6, the proposed antenna provides UWB characteristics from 3.1 GHz to 11.76 GHz. A 320.61% IBW improvement is obtained from step 1. The distribution of surface current at various frequencies is shown in Fig. 3.

**Table 2:** Frequency range (FR) and % of impedance bandwidth (BW) in various steps of the proposed MIMO.

Step	Band	$\:{\varvec{S}}_{11}$ (dB)	Frequency Range (GHz)	Bandwidth (BW) (GHz)	% of Impedance BW	BW ratio
Step-1	Single	−28.61	3.73–4.93	1.2	1.32:1	1.32:1
Step-2	Dual	−20.34 −26.05	8.56–10.3 8.96–11.43	0.4 1.13	1.04:1 1.11:1	1.04:1 1.11:1
Step-3	Single	−33.3	8.33–8.96	0.63	1.07:1	1.07:1
Step-4	Dual	−18.57 −13.45	8–8.96 11.03–11.76	0.96 0.73	1.12:1 1.07:1	1.12:1 1.07:1
Step-5	Dual	−21.50 −12.96	8.13–8.83 11.03–11.66	0.7 0.63	1.09:1 1.06:1	1.09:1 1.06:1
**Step-6**	**UWB**	**−23.08** **−29.50** **−20.70** **−20.34**	**3.1–11.76**	**8.66**	**3.79:1**	**3.79:1**

### Mathematical modelling

The following Eq. (1) to (4) is used for UWB CPW fed MIMO antenna^28^.

The dimensions of the slot are given by Eq. (1)

1 $$\:W=\:\frac{1}{2\times\:{f}_{r}\sqrt{\mu\:}\times\:{\epsilon}_{0}}\times\:\sqrt{\frac{2}{{\epsilon}_{r}+1}}$$

The substrate’s effective dielectric constant is determined using Eq. (3) as (2)

2 $$\:{\:\epsilon}_{reff}=\:\frac{{\epsilon}_{r}+1}{2}+\frac{{\epsilon}_{r}-1}{2}\times\:{\left[1+12\frac{h}{W}\right]}^{\frac{-1}{2}}$$

The increase in the antenna’s electrical size caused by the fringe effect is represented by the amount (ΔL). The extension length of the patch is determined by Eq. (3)

3 $$\:\frac{{\Delta\:}\text{L}}{h}=0.412\frac{\left({\epsilon}_{reff}+0.3\right)\left(\frac{W}{h}+0.264\right)}{\left({\epsilon}_{reff}+0.258\right)\left(\frac{W}{h}+0.8\right)}\:\:$$

Equation 4 is used to determine the patch L’s length.

4 $$\:\:L=\frac{c}{2{f}_{r}\sqrt{{\epsilon}_{reff}}}-2{\Delta\:}\text{L}\:$$

Where, $\:\epsilon$_0_ is the free space permittivity, W is the width of the slot, f_r_ is the resonant frequency, $\:\epsilon$_r_ is the substrate dielectric constant, µ is the permeability of free space, ΔL is the extended length, h is the substrate height, c is the speed of light (3 × 10^8^ m/sec),

## Experimental outcome

**Fig. 4:**
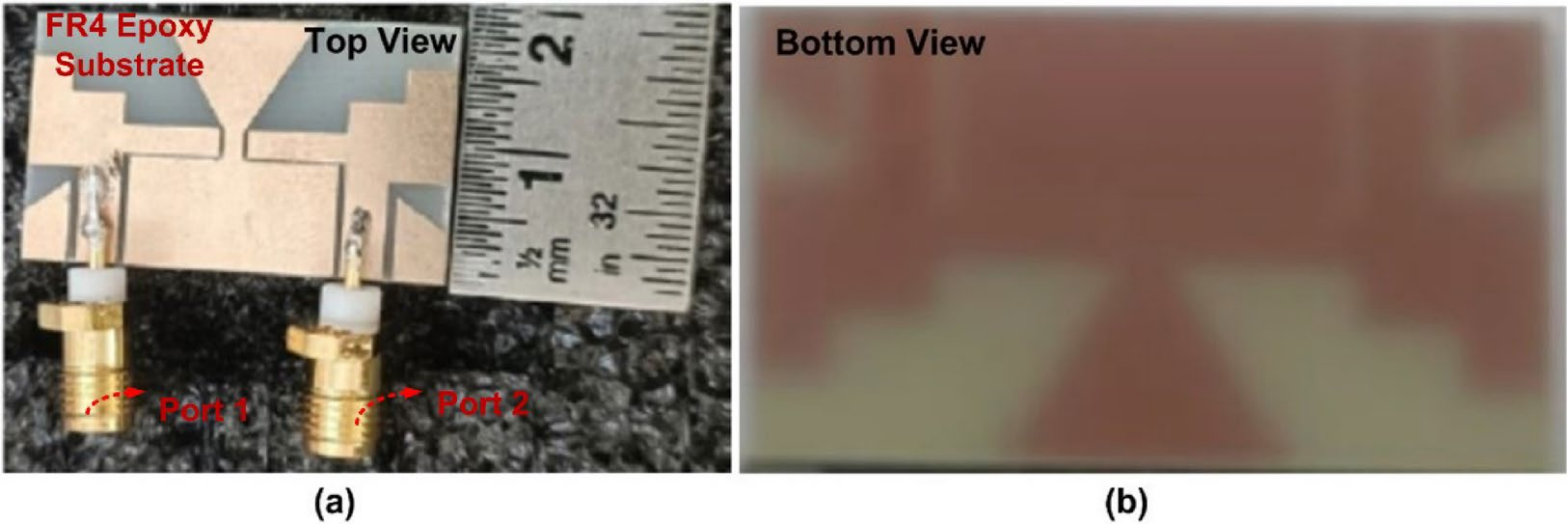
Fabricated suggested MIMO antenna.

**Fig. 5:**
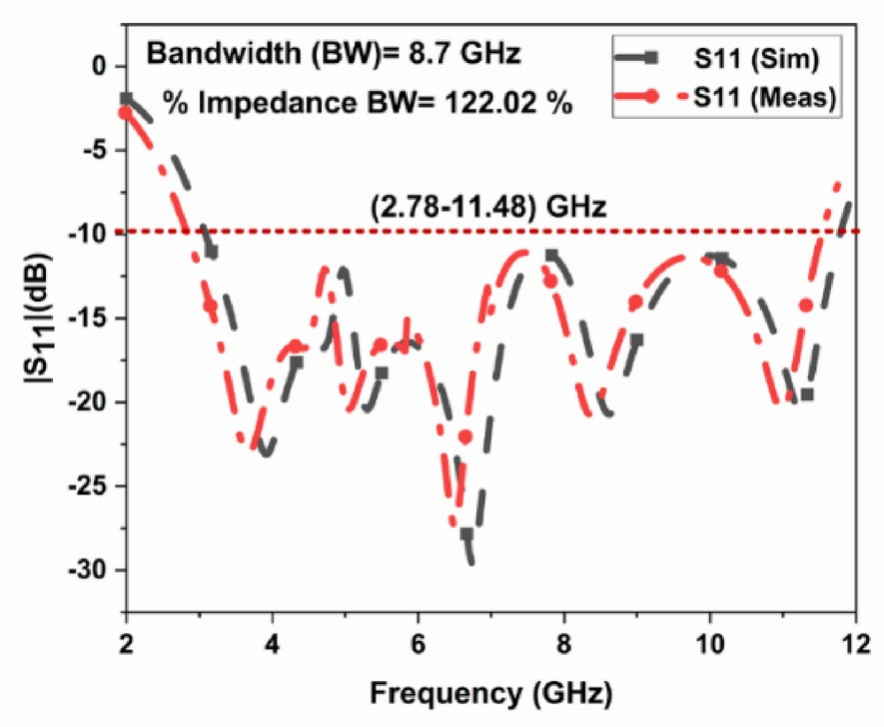
Measured, and simulated $\:{S}_{11}$ of the suggested MIMO antenna.

**Fig. 6:**
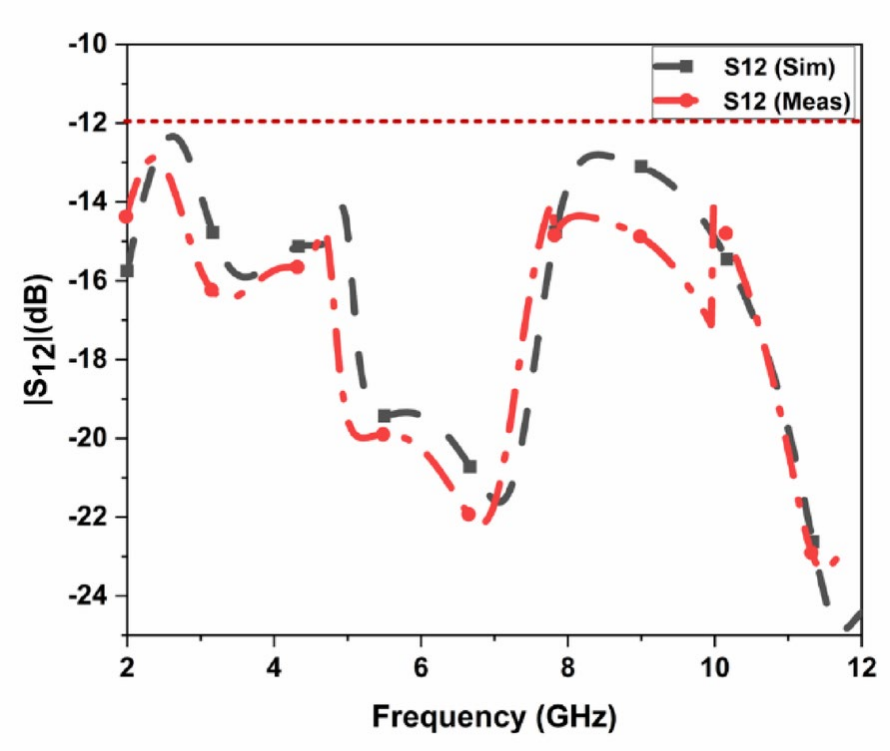
Measured, and simulated S12 of the suggested MIMO antenna.

**Fig. 7:**
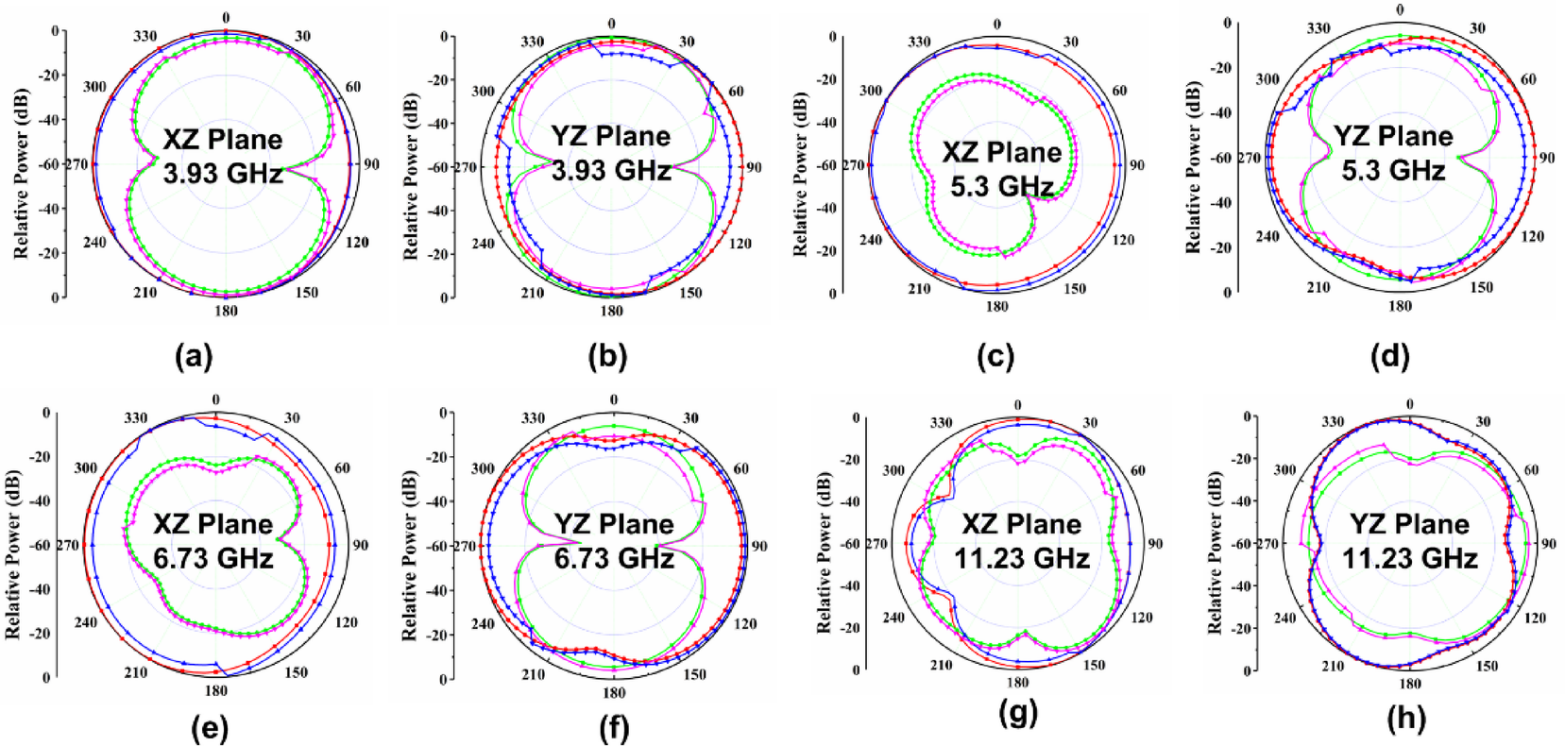
Measured, and simulated radiation patterns at various frequencies.

**Fig. 8:**
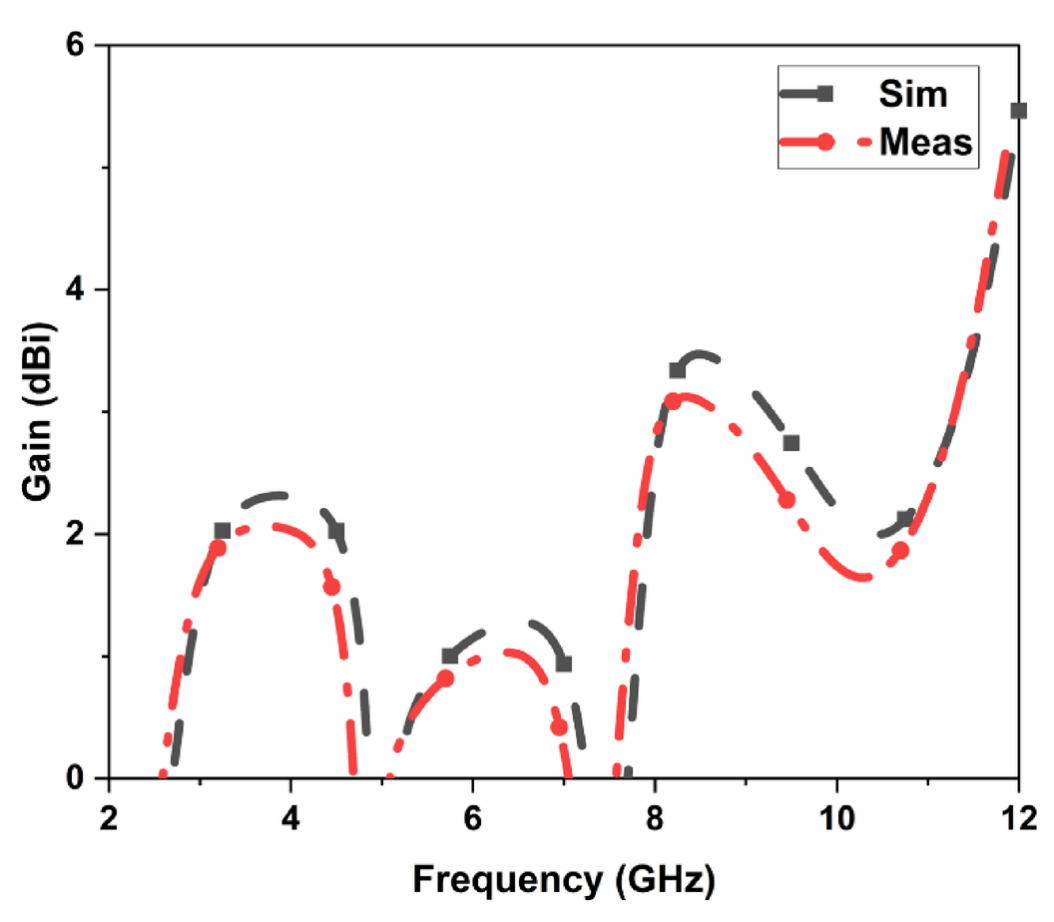
Simulated and Measured gain of the proposed CPW MIMO antenna.

The proposed CPW-fed MIMO antenna is fabricated to verify the simulated results, and it is shown in Fig. 4. The isolation $\:{S}_{12}$, reflection coefficient $\:{S}_{11}$, and radiation patterns are measured. The accuracy of the manufacturing and soldering are the reasons for the discrepancy between the simulated and measured outcomes. The simulated, and measured $\:{S}_{11}$ are illustrated in Fig. 5. The proposed antenna gives a simulated operating frequency from 3.1 GHz to 11.76 GHz. The measured $\:{S}_{11}$ provides an operating frequency from 2.78 GHz to 11.48 GHz. A bandwidth of 8.7 GHz is obtained. The percentage of impedance bandwidth is 122.02% is obtained. The proposed antenna is used for 5G New Radio Bands, Wi-Fi 5, DSRC, Wi-Fi 6, and V2 V, V2I, and V2 N in the operating band. The operating bands of n46 are 5.15 to 5.925 GHz, n47 is 5.855 to 5.925 GHz, n48 is 3.55 to 3.7 GHz, n77 is 3.3 to 4.2 GHz, n78 is 3.3 to 3.8 GHz, n79 is 4.4 to 5 GHz, and n96 is 5.925 to 7.125 GHz. The operating frequency range for V2 V, V2I, and V2 N is 5.85 to 5.925 GHz. The operating frequency range of Wi-Fi 5 and Wi-Fi 6 are 5.15 to 5.85 GHz and 5.925 to 7.125 GHz, respectively. The simulated, and measured isolation $\:{S}_{12}$ are illustrated in Fig. 6. The radiation patterns YZ Plane, and XZ Plane at various frequencies are shown in Fig. 7. Both planes demonstrate omnidirectional radiation patterns. The simulated, and measured gain of the proposed antenna are illustrated in Fig. 8.

## MIMO performance parameters

**Fig. 9:**
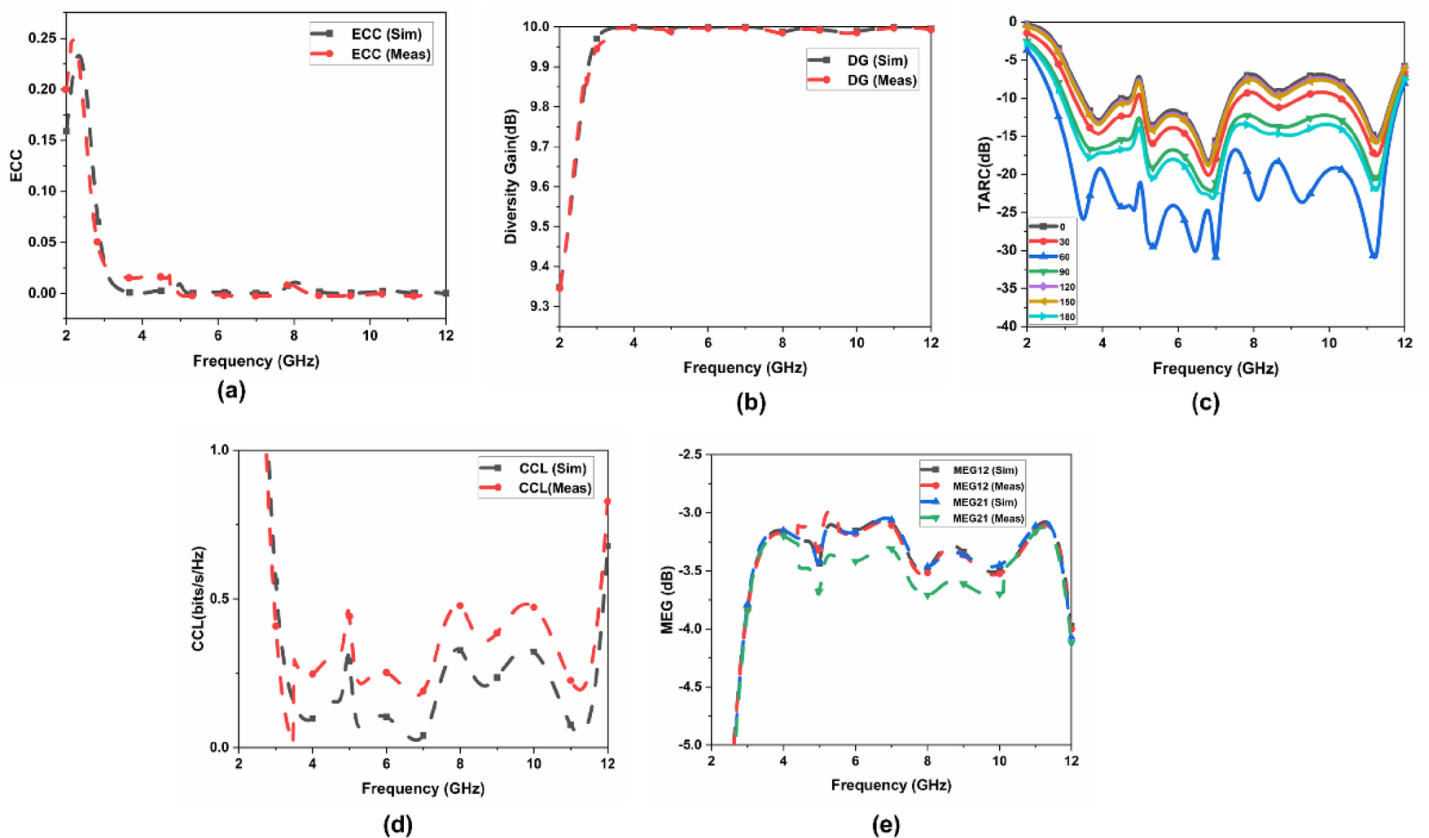
MIMO parameters (**a**) ECC, (**b**) DG, (**c**) TARC, (**d**) CCL, and (**e**) MEG.

The MIMO diversity performance is defined, as how efficiently two antennas are working individually. The diversity performance can be calculated using the diversity gain (DG), channel capacity loss (CCL), mean effective gain (MEG), envelope correlation coefficients (ECC), and total active reflection coefficients (TARC). The capacity to receive information individually from each antenna is given through ECC. To achieve better performance the value of ECC should be less than 0.5 and can be determined using the method used in^29^. The calculation of ECC, both experimentally and numerically using the antenna element’s radiation pattern (Eq. (5)) is an extremely complex process. If the antenna elements are properly matched and lossless, an alternative method of calculating ECC is to use S-parameters (Eq. (6)). The DG is calculated through Eq. (7). The effect of adjacent antenna components on each other when working together is given through TARC, and it is calculated through Eq. (8). The value of TARC should be < 0 dB for the MIMO communication. The value of TARC can be determined using the equation given in^30^. The CCL is calculated through Eq. (9)^31^. The MEG is calculated through Eq. (10)^32^. The MIMO parameters are shown in Fig. 9.

5 $$\:{ECC}_{12}=\:\frac{\left|\iint\:[\overrightarrow{{E}_{1}}\:\left(\theta\:,\phi\:\right)\overrightarrow{{E}_{2}}\left(\theta\:,\phi\:\right)d\varOmega\:\right|2}{\iint\:{\left|\overrightarrow{{E}_{1}\:}\right(\theta\:,\varphi\:\left)\right|}^{2}d\varOmega\:\iint\:{\left|\overrightarrow{{E}_{2}\:}\right(\theta\:,\varphi\:\left)\right|}^{2}d\varOmega\:}\:$$

Where, $\:\varOmega\:$ is the solid angle

6 $$\:\:{ECC}_{12}=\:\frac{{{{|S}^{*}}_{11\:}{S}_{12}+{{|S}^{*}}_{21\:}{S}_{22}|}^{2}}{(1-{{|S}_{11}|}^{2}-{{|S}_{21}|}^{2})(1-{{|S}_{22}|}^{2}-{{|S}_{12}|}^{2})}$$

7 $$\:DG=10\:\times\:\sqrt{1-{\left|ECC\right|}^{2}}$$

8 $$\:TARC=\:\frac{\sqrt{{{(S}_{11}+{S}_{12})}^{2}+{{(S}_{21}+{S}_{22})}^{2}}}{\sqrt{2}}$$

9 $$\:{C}_{loss}=-{log}_{2}\left|{\psi\:}^{R}\right|$$

The correlation matrix of the receiving antenna is defined through Eq. (10)

10 $$\:{\psi\:}^{R}=\left(\begin{array}{c}{\psi\:}_{11}\:\:\:\:\:{\psi\:}_{12}\\\:{\psi\:}_{21\:\:\:}\:{\psi\:}_{22}\end{array}\right)$$

The elements of the matrix are identified through Eqs. (11) and (12)

11 $$\:{\psi\:}_{ii}=1-({\left|{S}_{ii}\right|}^{2}+{\left|{S}_{ij}\right|}^{2})$$

12 $$\:{\psi\:}_{ij}=-({{S}^{*}}_{ii}{S}_{ij}+{{S}^{*}}_{ji}{S}_{jj})$$

Where *i = j* = 1 or 2.

Mean Effective Gain (MEG) is calculated through Eqs. (13) and (14)

13 $$\:{MEG}_{1}=\frac{[1-{\left|{S}_{11}\right|}^{2}-{\left|{S}_{12}\right|}^{2}]}{2}$$

14 $$\:{MEG}_{2}=\frac{[1-{\left|{S}_{21}\right|}^{2}-{\left|{S}_{22}\right|}^{2}]}{2}$$

## Optimization of the proposed antenna through machine learning

**Fig. 10:**
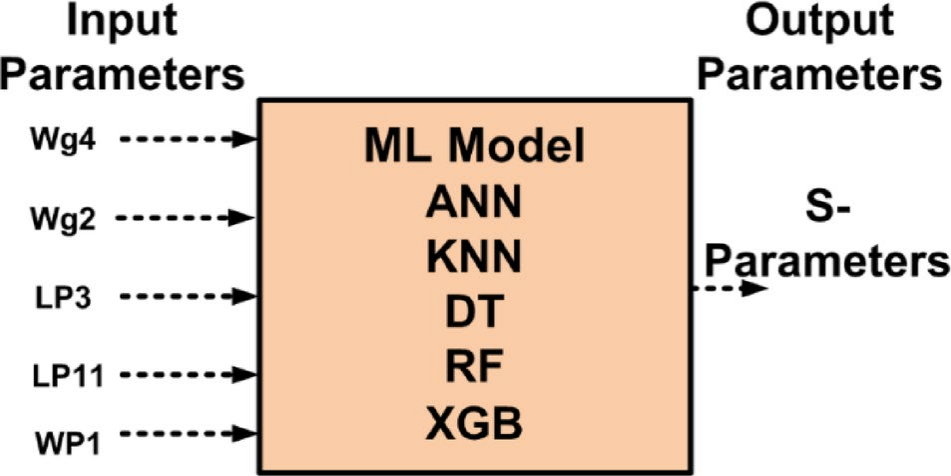
Input and output parameters of ML algorithms.

**Fig. 11:**
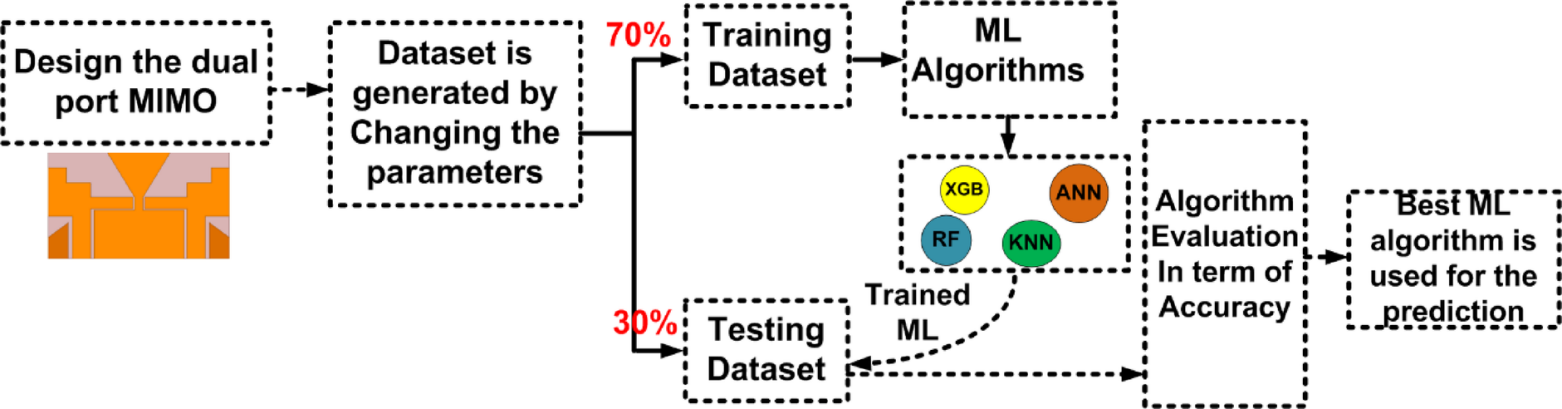
The flow chart of the ML in the suggested MIMO antenna.

**Fig. 12:**
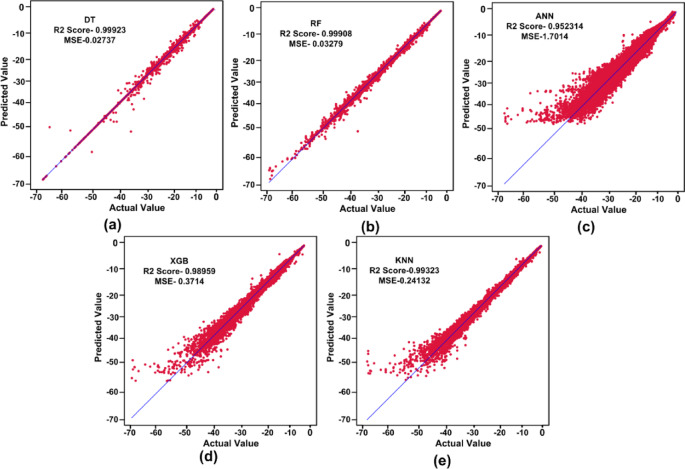
Actual vs. predicted values of various ML algorithms.

**Fig. 13:**
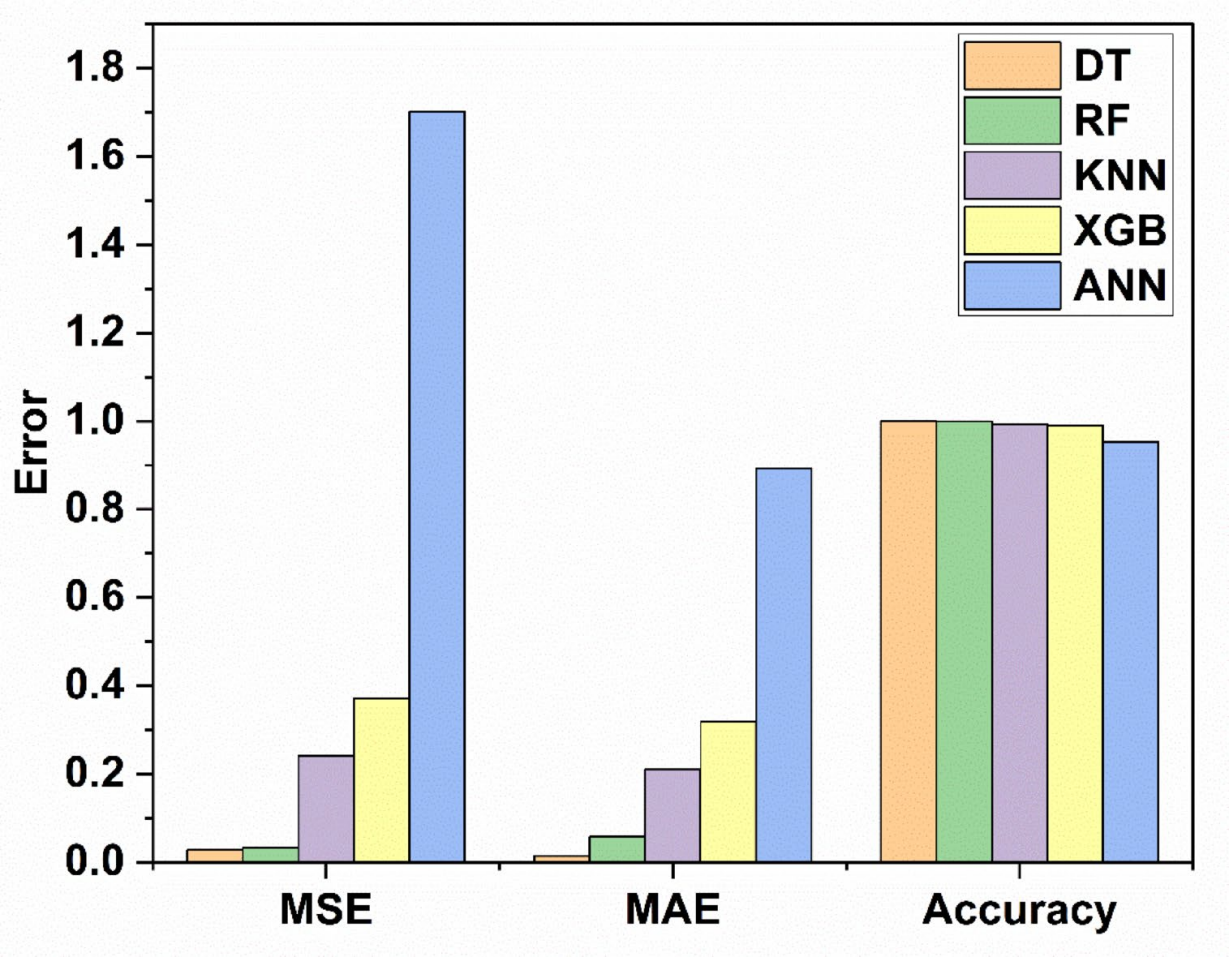
Error analysis of ML algorithms.

**Fig. 14:**
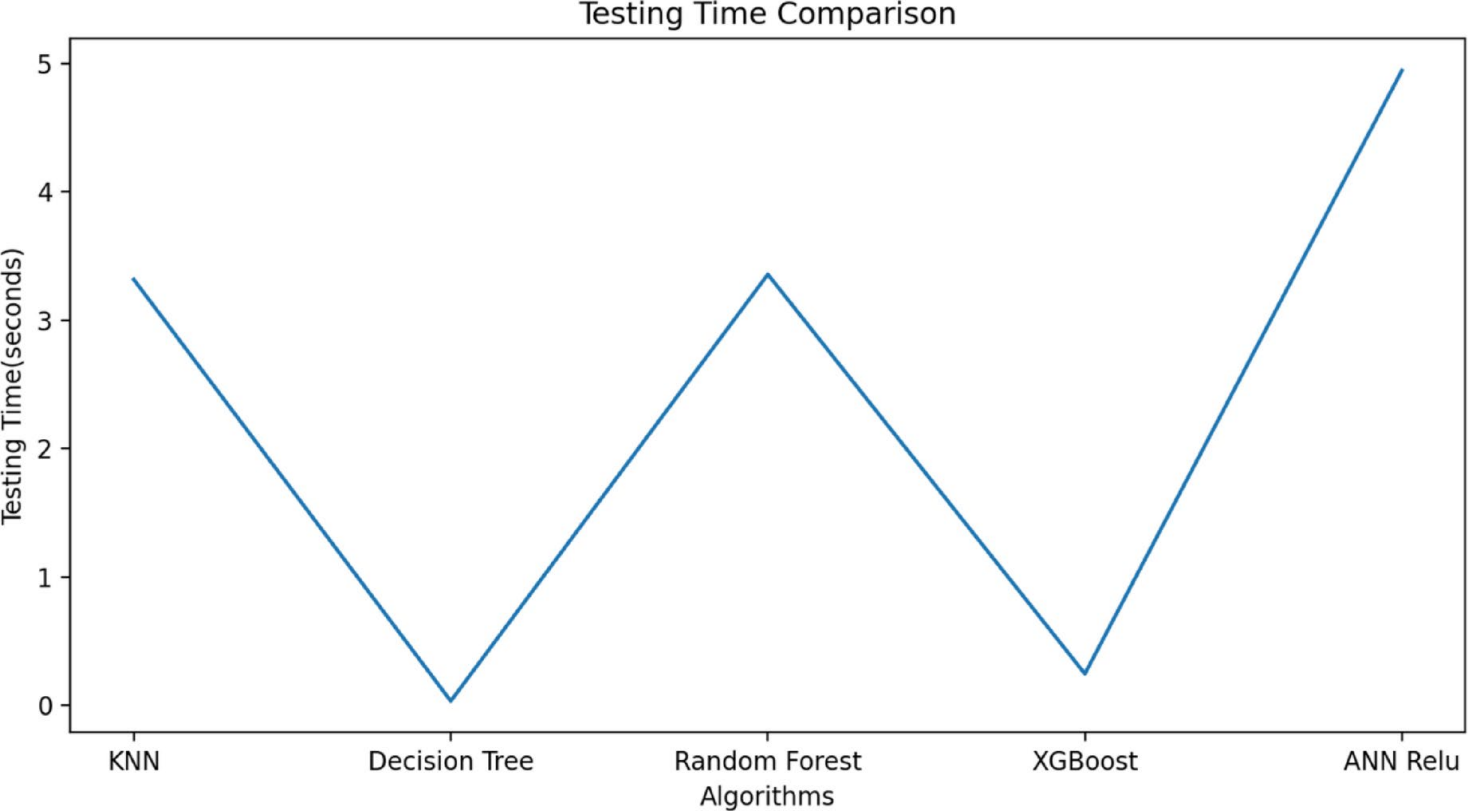
Testing time of ML algorithms.

**Fig. 15:**
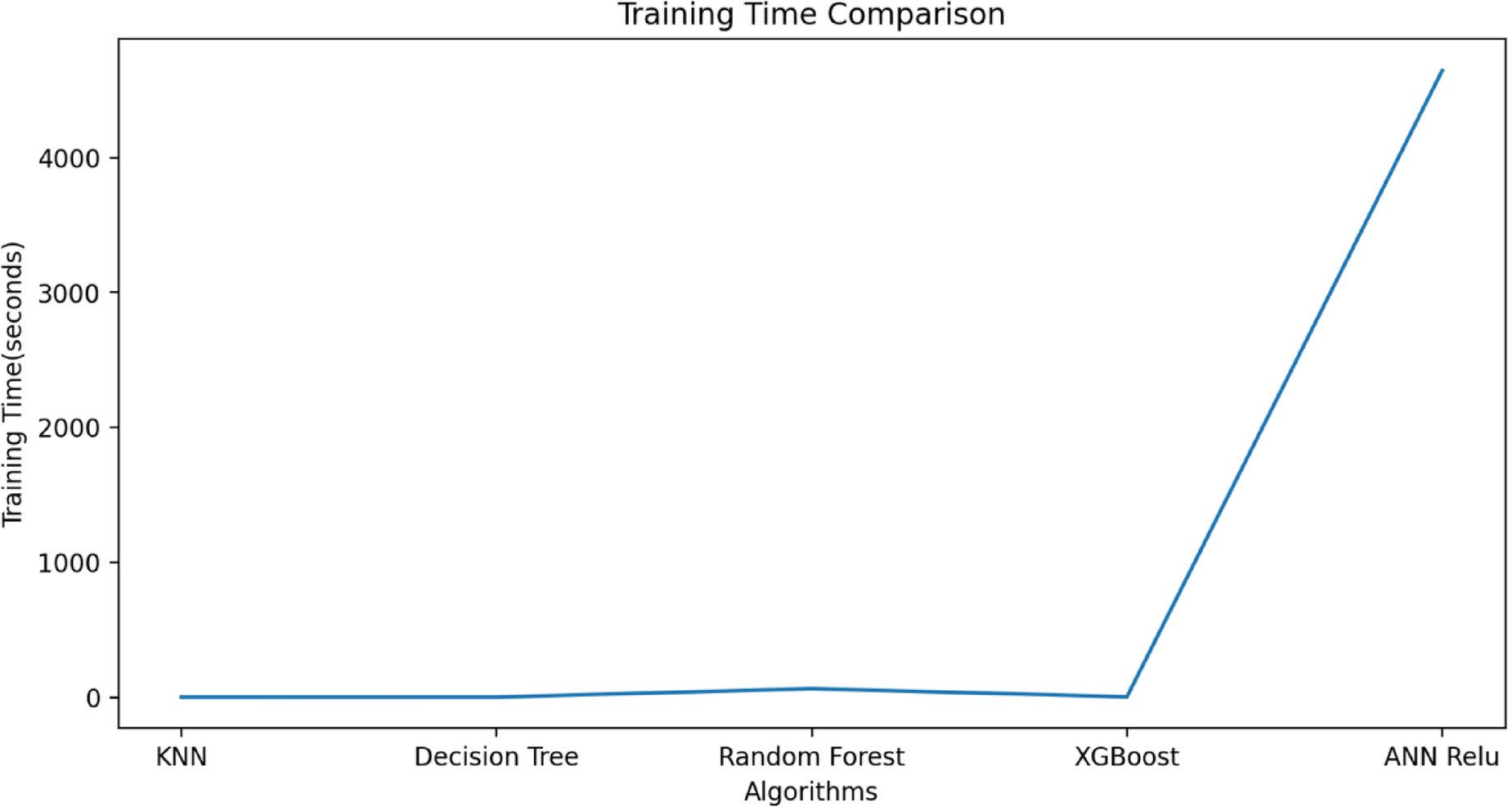
Training Time of ML algorithms.

**Fig. 16:**
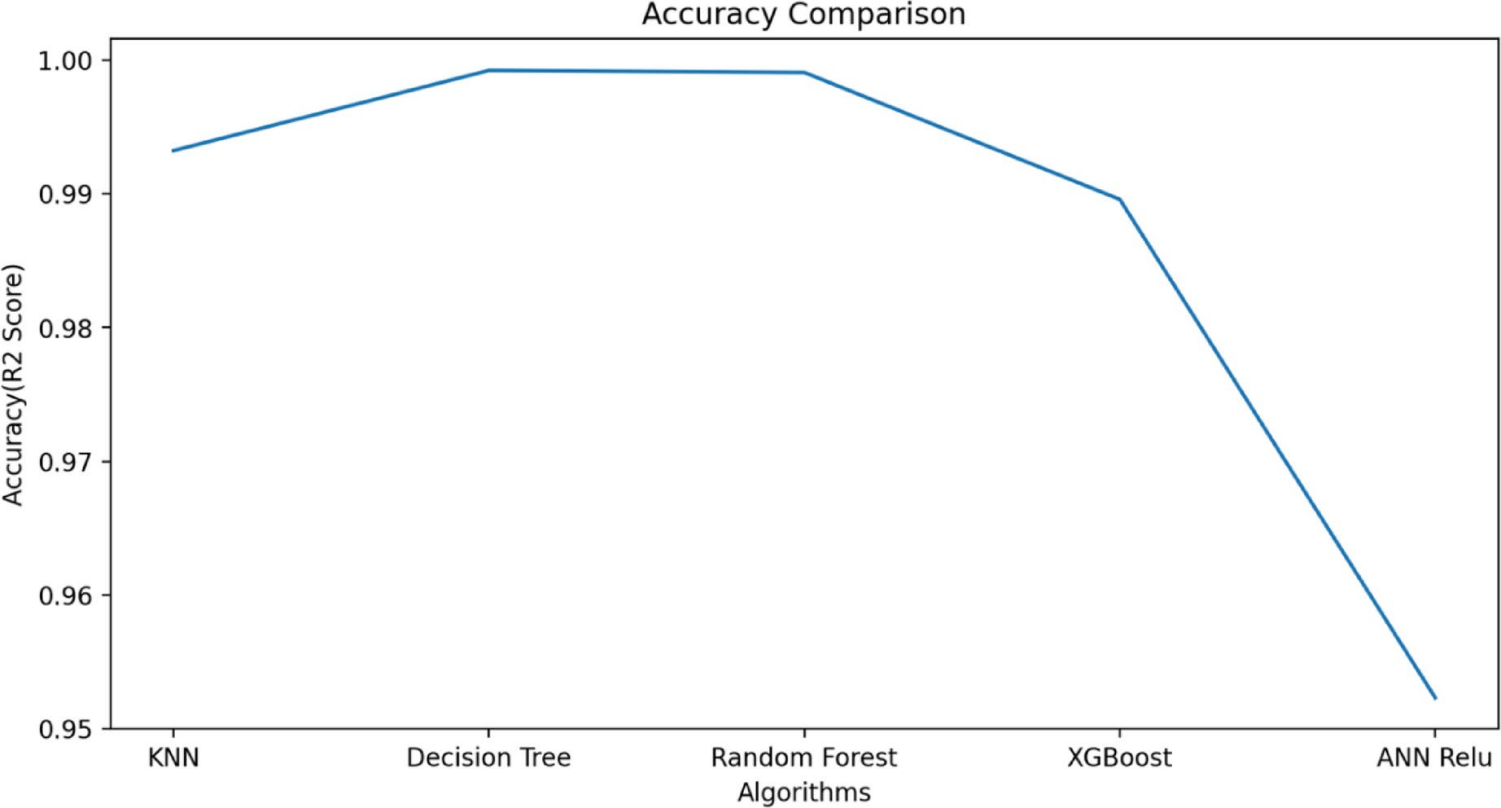
Accuracy of ML algorithms.

**Fig. 17:**
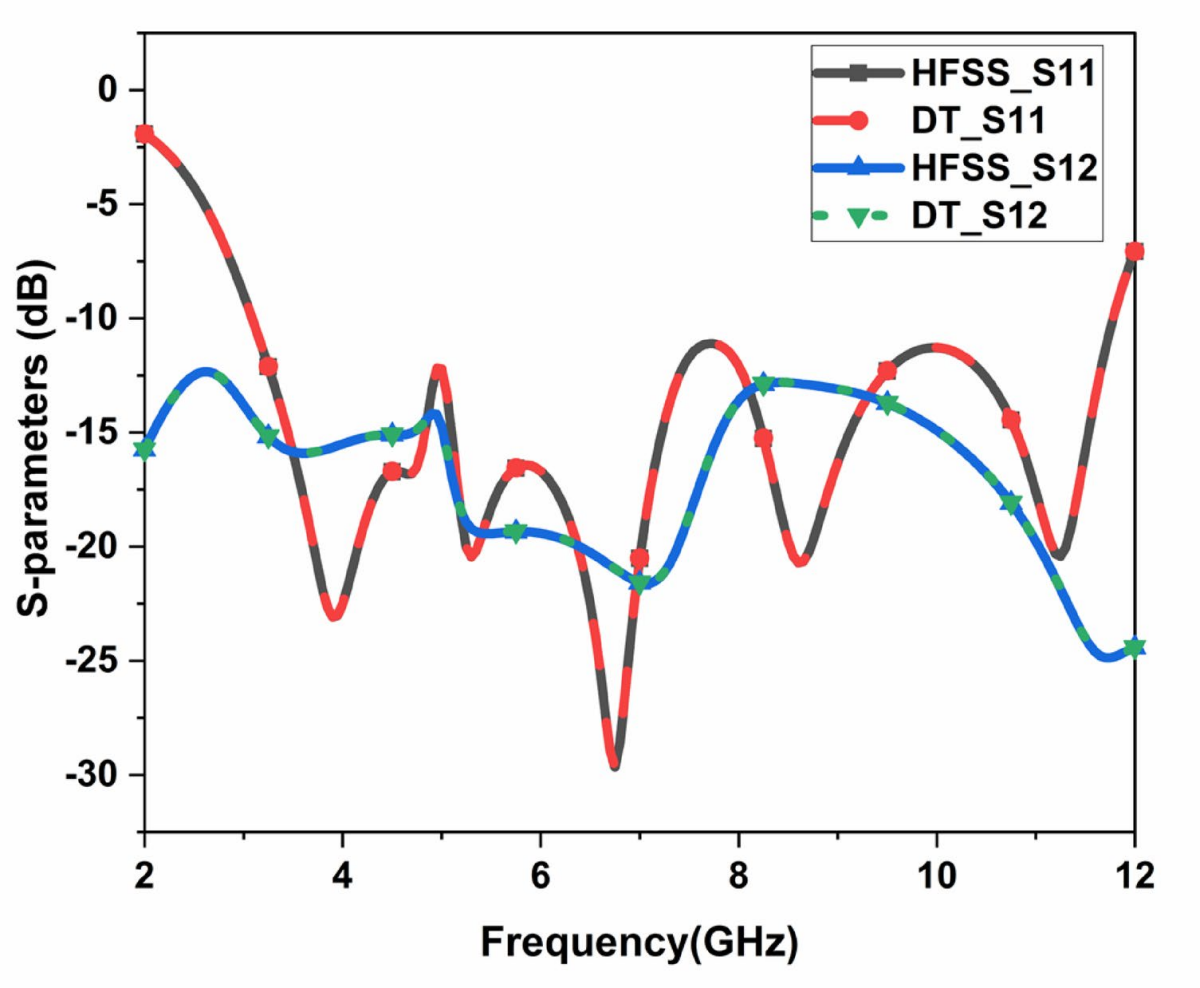
Comparison of various ML Algorithms with HFSS.

ML algorithm is used to predict the S_11_ and S_12_of the proposed antenna. Choosing the ML technique will reduce the number of simulations, saving a lot of time and error rates^33–38^. The performance of the proposed antenna depends on the design parameters, $\:{W}_{g4}$, $\:{W}_{P1}$,$\:{L}_{P11}$ $\:{L}_{P3}$and$\:\:{W}_{g2}$. These variables act as input for ML models as illustrated in Fig. 10. The $\:{W}_{g4}\:$varies from 1.3 to 1.5 mm with a step size of 0.1 mm, The $\:{W}_{P1}\:$varies from 2 to 3 mm with a step size of 0.25 mm, The $\:{W}_{g2}\:$varies from 14 to 15 mm with a step size of 0.1 mm, and the $\:{L}_{P3}\:$varies from 7 to 8 mm with a step size of 0.25 mm is used to generate the dataset from the HFSS and used for training the ML models. 70% of the dataset is used for training the model, and 30% dataset is used for testing purposes. The ML flow chart for the suggested antenna is illustrated in Fig. 11^33–36^. To achieve better results, it depends on the dataset. The Mean Square Error (MSE), Mean Absolute Error (MAE), and R^2^ Score are given in Table 3. Actual vs. predicted values for ML algorithms are shown in Fig. 12. The error analysis of ML algorithms is illustrated in Fig. 13. The training time, testing time, and accuracy of ML algorithms are illustrated in Figs. 14 and 15, and 16. and Fig. 17. shows the comparison of various ML Algorithms with HFSS.

**Table 3:** MSE, MAE and R^2^ scores of different ML algorithm.

ML Algorithm	MSE	MAE	*R*^2^ score
DT	0.02732	0.0139	0.99923
RF	0.03279	0.0575	0.99908
KNN	0.24137	0.21017	0.99323
XGB	0.3714	0.3182	0.98959
ANN	1.7014	0.8933	0.95231

## Conclusion

In this work, a very compact CPW Fed ladder-shaped MIMO antenna for UWB application is presented. A bandwidth of 8.7 GHz is achieved with an isolation of less than − 12 dB. The antenna shows stable radiation patterns. The proposed antenna is optimized using the ML models to reduce the simulation time. The DT ML model achieved the best accuracy of 99.92% for the prediction of S-parameters. Hence the suggested MIMO antenna is suitable for next-generation wireless communication applications.

## Data Availability

The datasets used and/or analyzed during the current study available from the corresponding author on reasonable request.
